# Women's Preferences for Masculinity in Male Faces Are Predicted by Material Scarcity, But Not Time or Psychological Scarcity

**DOI:** 10.1177/14747049231175073

**Published:** 2023-09-21

**Authors:** Anthony J. Lee, Nikita K. J. McGuire

**Affiliations:** Faculty of Natural Sciences, Division of Psychology, 7622 University of Stirling, Stirling, Scotland

**Keywords:** attraction, mate preference, sexual dimorphism, resource scarcity, perceived scarcity, individual differences

## Abstract

Facial femininity in men is purportedly used as a cue by women as a signal of parental quality and willingness to provide resources. Accordingly, in contexts where choosing a partner that will provide resources is more beneficial (e.g., when resources are scarce), women have shown an increase preference for facial femininity in male faces. However, domains of scarcity often covary, and it is, therefore, unclear whether these contextual shifts in facial masculinity/femininity preferences are specific to material scarcity (as implied by previous theory), or due to an unrelated domain of scarcity (e.g., time or psychological scarcity). Here, a sample of 823 women completed the Perceived Scarcity Scale, which measures three separate domains of scarcity: material scarcity, time scarcity, and psychological scarcity. Participants also rated the attractiveness of 42 male faces, which were measured on objective sexual dimorphism and perceived masculinity. Consistent with theory, material scarcity, and not time or psychological scarcity, was associated with a decreased preference for objective sexual dimorphism (i.e., an increased preference for facial femininity). This study provides evidence that women use sexual dimorphism as a cue to material resource provisioning potential when assessing men as a mate.

## Introduction

Women purportedly face a trade-off between good health and good parental quality when choosing a male partner. Facial sexual dimorphism (e.g., the masculinity of male faces) is theorised to be associated with health and disease resistance (Rantala et al., 2012; Rhodes et al., 2003; Thornhill & Gangestad, 2006; but see Boothroyd et al., 2013). As such, it is proposed that women should show a preference for facial sexual dimorphism in men as these mates may incur benefits to their own fitness, either directly (e.g., through decreased exposure to pathogens) or indirectly (i.e., genetic health benefits inherited by offspring, Gangestad & Simpson, 2000; but see Lee et al., 2014). However, facial masculinity in men is also associated with poor parental quality; facial masculinity in men is associated with higher mating success (Hill et al., 2013; Kordsmeyer et al., 2018) and a greater preference for short-term relationships (Boothroyd et al., 2008). Men with masculine faces also report being less likely to be faithful in a relationship (Boothroyd et al., 2008; Rhodes et al., 2005; Rhodes et al., 2013), and are generally perceived as less paternally investing (Boothroyd et al., 2007; Kruger, 2006). As such, when choosing a partner, women face a trade-off between choosing a facially masculine man with good health, or a more feminine man that may better provide resources to future offspring (Gangestad & Simpson, 2000; Holzleitner & Perrett, 2017).

Indeed, in contexts where having a healthy partner is more beneficial, women have been found to show an increase preference in male facial sexual dimorphism. For instance, increased preference for facial masculinity is associated with lower local health indices (DeBruine et al., 2011; DeBruine, Jones, Crawford, et al., 2010). Experimental studies have found that increased facial masculinity preferences are associated with perceived exposure to pathogens (Little et al., 2011). There is also evidence that individual differences in pathogen disgust sensitivity is also associated with women's preferences for facial masculinity (DeBruine, Jones, Tybur, et al., 2010; Lee et al., 2013; Lee & Zietsch, 2015). However, note there is a growing body of literature that have failed to replicate these findings (Marcinkowska et al., 2019; McIntosh et al., 2017; Saribay et al., 2021; Tybur et al., 2022).

Conversely, in contexts where choosing a committed partner willing to invest and provide resources to potential offspring is more beneficial, women have been shown to have an increased preference for facial femininity. Indeed, experimental evidence suggests that women report a greater preference for facial femininity when primed with resource scarcity or environmental harshness (Little et al., 2007; Little et al., 2012; Lyons et al., 2016; Watkins et al., 2012). Also, greater preferences for facial femininity is associated with individual differences in SES or perceived financial hardship (Holzleitner & Perrett, 2017; Lee et al., 2013). More generally, the influence of resource scarcity appears to generalise to non-face preferences for traits purportedly associated with resource provisioning potential (Bradshaw et al., 2020; Lee et al., 2015; Lee & Zietsch, 2011; Marzoli et al., 2013; Nelson & Morrison, 2005; Pisanski & Feinberg, 2013). Similarly, previous research has proposed that women report a greater preference for facial femininity for long-term relationships compared to short-term relationships, where choosing a partner willing to invest is of greater importance (Clarkson et al., 2020; Little & Hancock, 2002; Little & Jones, 2012; Penton-Voak et al., 2003). However, the literature here is mixed as other studies have found a greater preference for masculine traits for long-term attractiveness (Dixson et al., 2018; Dixson et al., 2019; Dixson & Brooks, 2013; Stower et al., 2020).

When investigating effects of resource scarcity on mate choice, previous research often implies the effects are due to material scarcity (i.e., women show a greater preference for feminine male faces as these men are more likely to provide material resources beneficial for survival). However, material scarcity often covaries with other socio-economic factors, including other dimensions of scarcity. For instance, individuals who experience resource scarcity often face additional time pressures (time scarcity), or lack interpersonal or intrapersonal resources (e.g., social support, self-efficacy; psychological scarcity). Indeed, conceptualising scarcity comprising of several dimensions has been useful in the field of health psychology for accurately predicting perceived stress, health, quality of life, and mental health (DeSousa et al., 2018; DeSousa et al., 2020; DeSousa & Rego, 2022). Applying this model of scarcity to research investigating mate choice may provide insight into the exact selection pressures that may motivate shifts in preferences, and whether previously identified effects for facial masculinity preferences are specific to material scarcity (and therefore reinforcing the theory that sexual dimorphism is used as a cue for resource provisioning potential), or potentially due to another domain of scarcity, or scarcity more generally.

Here, we assess the influence of resource scarcity on women's preference for facial masculinity using the Perceived Scarcity Scale (DeSousa et al., 2020), which separates scarcity into three domains: material scarcity, time scarcity, and psychological scarcity. According to previous theory, we hypothesise that increased material scarcity, but not time scarcity or psychological scarcity, would be negatively associated with facial masculinity preferences.

## Methods

### Participants

Participants were 823 volunteers who completed the online study recruited via posts on social media. Participants were removed from the sample if they did not report being female (n  =  4) or did not report being attracted to men (n  =  17). This resulted in a final sample of 802 women (*M*  =  24.75 years, *SD*  =  7.87 years). 35.08% of participants reported being single, 62.30% reported being in a committed relationship, while the remainder reported other.

### Measures

*Perceived Scarcity Scale*. The Perceived Scarcity Scale (DeSousa et al., 2020) measures scarcity on three domains: material, time, and psychological scarcity. Participants rated their agreement to statements on a 7-point scale (1  =  strongly disagree, 7  =  strongly agree). The material scarcity subscale includes eight items, including “I have had my utilities (e.g., heat, water, etc.) turned off because I could not pay my bill”. The time scarcity subscale also includes eight items, such as “I have enough time to engage in hobbies or engage in activities I enjoy” (reverse-coded). The psychological scarcity subscale includes 8 items, including “There are people in my life I can go to for support when I need it” (reverse-coded). Scores for each subscale were calculated separately such that higher scores indicated greater scarcity in that domain. The order that the items were shown was randomised for each participant.

*Face Rating Task.* Participants were presented with male faces sequentially and asked to rate how attractive they found them on a 9-point scale (1  =  Very Unattractive, 9  =  Very Attractive). Participants were presented with 42 male faces in a randomised order. For each face, two facial metrics scores were calculated: objective facial sexual dimorphism, and perceived masculinity. To calculate objective facial sexual dimorphism, we used the vector methods used in previous research (e.g., Holzleitner et al., 2014; Komori et al., 2011; Valenzano et al., 2006) using functions from the facefuns R package (Holzleitner & DeBruine, 2021). This involved using geometric morphometric techniques to extract the face shape information from 155 landmarks delineated on all male and female faces in the Chicago Face Database (Ma et al., 2015). Objective sexual dimorphism is then calculated by computing a multi-dimensional vector between the average female and average male face shapes from this sample, and then projecting each male face included in our study onto this vector. This produces a single score for each male face representing its position along the male–female face shape continuum. Scores are scaled such that higher scores indicate more male-like faces. Perceived masculinity scores were taken from the norming data accompanying the Chicago Face Database. As reported in Ma et al. (2015), trait ratings (including masculinity) were based on the average judgement of 1087 raters who rated each face on a 7-point scale (e.g., 1  =  not at all masculine, 7  =  extremely masculine). Raters were instructed to consider each target in relation to others of the same race and gender when making each judgement. Consistency among raters judging masculinity was high (Cronbach's alpha  =  .99; Ma et al., 2015). For each participant, their preference for facial masculinity can be determined by comparing the facial metric scores (either objective sexual dimorphism or perceived masculinity) with their ratings of attractiveness.

### Procedure

The Perceived Scarcity Scale and the Face Rating Task were included in a larger online survey investigating mate preferences. The order in which participants viewed each task was randomised.

### Statistical Analysis

The data was analysed using mixed effects modelling and conducted in the R statistical software (R Core Team, 2013) using the lme4 (Bates et al., 2015) and lmerTest (Kuznetsova et al., 2015) packages. Separate models were conducted for objective facial sexual dimorphism and perceived facial masculinity. For both models, fixed effects estimates were included for material scarcity, time scarcity, and psychological scarcity, which were all z-standardised at the participant level. Predictors also included facial metrics scores (either objective sexual dimorphism or perceived masculinity), which were z-standardised at the stimulus level, as well as their interaction with material, time, and psychological scarcity. For the hypothesis to be supported, we would expect a significant interaction between the facial metric scores and material scarcity (but not time nor psychological scarcity) such that preference for facial masculinity decreases when material scarcity increases. The models included a crossed random effect structure (DeBruine & Barr, 2021) with grouping factors for participant ID and stimulus ID. Random intercepts and slopes were specified maximally, according to Barr et al. (2013) and Barr (2013). The fixed effects estimate for each model are reported here; for full model specification and results, including random effects, see the supplementary materials on the OSF (https://osf.io/bwvzu/).

## Results

Correlations between the Perceived Scarcity Scale subscales indicated that multicollinearity was not an issue (*r*s < .43). The fixed effects estimates for models including objective sexual dimorphism and perceived masculinity are reported in Table 1 and Table 2 respectively. Across both models, the only significant effect was the interaction between objective sexual dimorphism and material scarcity in the predicted direction – as material scarcity increased, preference for facial sexual dimorphism in male faces decreased. It should be noted that, while not significant, the interaction between perceived masculinity and material scarcity was trending in the same direction. No other main effect or interaction was significant.

**Table 1.** Fixed Effects Estimates from the Objective Sexual Dimorphism Model Predicting Attractiveness Ratings.

	Estimate (Std. Error)	t-value (approx. df)	p-value
Intercept	3.01 (.09)	34.81 (127.04)	< .001 ***
Objective Sexual Dimorphism	.00 (.08)	.03 (55.81)	.973
Material Scarcity	.05 (.04)	1.16 (811.99)	.248
Time Scarcity	−.01 (.04)	−.26 (799.59)	.797
Psychological Scarcity	−.00 (.05)	−.04 (801.60)	.970
Sexual Dimorphism × Material Scarcity	−.02 (.01)	−2.48 (48.68)	.017*
Sexual Dimorphism × Time Scarcity	.01 (.01)	1.23 (107.62)	.222
Sexual Dimorphism × Psychological Scarcity	−.00 (.01)	−.16 (54.97)	.871

* *p* < .05, ** *p* < .01, *** *p* < .001

**Table 2.** Fixed Effects Estimates from the Perceived Masculinity Model Predicting Attractiveness Ratings.

	Estimate (Std. Error)	t-value (approx. df)	p-value
Intercept	3.01 (.08)	35.99 (121.91)	< .001 ***
Perceived Masculinity	.10 (.07)	1.58 (14.78)	.135
Material Scarcity	.05 (.04)	1.16 (811.17)	.246
Time Scarcity	−.01 (.04)	−.25 (798.51)	.801
Psychological Scarcity	−.00 (.04)	−.03 (800.24)	.973
Masculinity × Material Scarcity	−.02 (.01)	−1.95 (76.47)	.055
Masculinity × Time Scarcity	.01 (.02)	.84 (205.04)	.403
Masculinity × Psychological Scarcity	−.01 (.01)	−1.47 (85.44)	.146

* *p* < .05, ** *p* < .01, *** *p* < .001

## Discussion

As hypothesised, we found that only material scarcity, and not time or psychological scarcity, was negatively associated with preference for facial sexual dimorphism. This is consistent with the previous assumption that facial femininity in men is used as a cue to resource provisioning potential (Boothroyd et al., 2007, 2008; Kruger, 2006; Rhodes et al., 2005, 2013). These findings provides insight into the selection pressures underlying previous findings that women report an increase preference for facial femininity in contexts of resource scarcity (Holzleitner & Perrett, 2017; Lee et al., 2013; Little et al., 2007, 2012; Lyons et al., 2016; Watkins et al., 2012), and suggest that these effects are likely driven by material scarcity and not scarcity more generally. Our results also do not support possible alternative explanations; for example, in harsh environments, women may experience greater psychological scarcity and perhaps show a greater preference for facial femininity as feminine men are often perceived as being warm and compassionate (Buckingham et al., 2006; Wen et al., 2020).

Despite our findings being consistent with the predominant theory, there is a growing body of literature that has not replicated the association between resource scarce ecological conditions and women's facial sexual dimorphism preferences (Batres & Perrett, 2016; Dixson et al., 2017; Marcinkowska et al., 2019; Saribay et al., 2021). There has also been the suggestion that in some circumstances (e.g., environments where intrasexual competition is high) masculine men may be preferred more as they are more able to procure resources (Brooks et al., 2011; Little et al., 2012; Mefodeva et al., 2020; Puts, 2010). The results from this study may help reconcile this divergent literature – isolating material scarcity, rather than using broader indices of socio-economic position or manipulations where separate domains of scarcity are likely to confounded, may help better identify the effect.

Added to the complication is that the observed effects from our study are small - the change in marginal R^2^ (i.e., the proportion variance in attractiveness ratings explained by the fixed effects of the model) between objective sexual dimorphism models with and without material scarcity is small (Δ marginal R^2^  =  .002). This raises the question on whether such an effect is biologically meaningful; that is, is the effect likely to have a meaningful impact on the phenotype. Particularly since other factors, such as genetics, have been shown to have a much larger influence on women's preference for facial masculinity (Zietsch et al., 2015).

Another caveat to consider is the levels of resource scarcity experienced by participants in our study. While we found good variability on the material scarcity subscale of the Perceived Scarcity Scale (the full range of the scale was represented in the sample), the distribution was skewed such that much of the sample reported low material scarcity. This could be expected, as participants, at a minimum, required Internet access to participate in the study, and the sample likely was recruited predominately from a Western population. As such, including a wider experience of material scarcity would produce results that would better generalise to other populations. Indeed, some evidence suggests that resource scarcity may not impact preference in non-Western populations (Saribay et al., 2021).

One advantage of our study is that we distinguish between objective sexual dimorphism and perceived masculinity. This is potentially important as the research literature to this point often considers these interchangeable. While there was a positive correlation between the two in our stimuli set (*r*  =  .53), we found that the estimated material scarcity coefficients were of similar magnitudes, even though the effect for objective sexual dimorphism was significant while the effect for perceived masculinity was not.

In conclusion, our results suggest that previous results of resource scarcity moderating women's preference for facial masculinity is specific to material scarcity. While this finding is consistent with previous theories, there are still unanswered questions that future research should address.

## Supplemental Material

sj-csv-1-evp-10.1177_14747049231175073 - Supplemental material for Women's Preferences for Masculinity in Male Faces Are Predicted by Material Scarcity, But Not Time or Psychological Scarcity Click here for additional data file. Supplemental material, sj-csv-1-evp-10.1177_14747049231175073 for Women's Preferences for Masculinity in Male Faces Are Predicted by Material Scarcity, But Not Time or Psychological Scarcity by Anthony J. Lee and Nikita K. J. McGuire in Evolutionary Psychology

sj-csv-2-evp-10.1177_14747049231175073 - Supplemental material for Women's Preferences for Masculinity in Male Faces Are Predicted by Material Scarcity, But Not Time or Psychological Scarcity Click here for additional data file. Supplemental material, sj-csv-2-evp-10.1177_14747049231175073 for Women's Preferences for Masculinity in Male Faces Are Predicted by Material Scarcity, But Not Time or Psychological Scarcity by Anthony J. Lee and Nikita K. J. McGuire in Evolutionary Psychology

sj-Rmd-3-evp-10.1177_14747049231175073 - Supplemental material for Women's Preferences for Masculinity in Male Faces Are Predicted by Material Scarcity, But Not Time or Psychological Scarcity Click here for additional data file. Supplemental material, sj-Rmd-3-evp-10.1177_14747049231175073 for Women's Preferences for Masculinity in Male Faces Are Predicted by Material Scarcity, But Not Time or Psychological Scarcity by Anthony J. Lee and Nikita K. J. McGuire in Evolutionary Psychology
